# Smoking patterns and sociodemographic factors associated with tobacco use among Chinese rural male residents: a descriptive analysis

**DOI:** 10.1186/1471-2458-8-248

**Published:** 2008-07-21

**Authors:** Tingzhong Yang, Fuzhong Li, Xiaozhao Yang, Zhenyi Wu, Xiangxian Feng, Yibo Wang, Xuhui Wang, Abu Saleh M Abdullah

**Affiliations:** 1 Center for Tobacco Control Research, Zhejiang University School of Medicine, Yuhangtang Road, Hangzhou 310058, PR China; 2 Oregon Research Institute, Eugene, Oregon, USA; 3 Shanxi Health Inspection Bureau, Taiyuan, PR China; 4 Changzhi Medical College, Changzhi, PR China; 5 Xuzhou Medical College, Xuzhou, PR China; 6 Guizhou Nursing College, Guiyang, PR China; 7 Department of International Health, Boston University School of Public Health, Boston, Massachusetts, USA

## Abstract

**Background:**

Although evidence has shown high prevalence rates of tobacco use in the general urban populations in China, relatively little is known in its rural population. The purposes of this study were to examine smoking patterns and sociodemographic correlates of smoking in a sample of rural Chinese male residents.

**Methods:**

The study employed a cross-sectional, multi-stage sampling design. Residents (N = 4,414; aged 15 years and older) were recruited from four geographic regions in China. Information on participants' tobacco use (of all forms), including their daily use, and sociodemographic characteristics were collected via survey questionnaires and the resultant data were analyzed using chi-square tests and logistic regression procedures.

**Results:**

The overall smoking prevalence in the study sample was 66.8% (n = 2,950). Of these, the average use of tobacco products per day was 12.70 (SD = 7.99) and over 60% reported daily smoking of more than 10 cigarettes. Geographic regions of the study areas, age of the participants, marital status, ethnicity, education, occupation, and average personal annual income were found to be significantly associated with an increased likelihood of smoking among rural Chinese male residents.

**Conclusion:**

There is a high smoking prevalence in the Chinese rural population and smoking behaviors are associated with important sociodemographic factors. Findings suggest the need for tobacco control and intervention policies aimed at reducing tobacco use in Chinese rural smoking populations.

## Background

Tobacco use is a significant public health concern worldwide and one of the major causes of death and disability in both developed and developing countries. Based on World Health Organization (WHO) statistics, each year about 4 million deaths are attributable to tobacco use, and approximately 70% of those deaths occur in developing countries. The number of tobacco-related deaths is expected to rise to 8.4 million by 2020 [1]. China leads the world both in consumption of tobacco and in smoking-related deaths. Large-scale epidemiological studies have shown that smoking was responsible for approximately 1 million deaths per year in China during the 1990's [2,3]. The number is projected to reach 2 million per year by 2025 and increase to about 3 million by 2050. Thus, a total of 100 million Chinese will die from the effects of smoking or have smoking-related deaths in the next 50 years if the current smoking rate continues [4].

China is largely a country of rural agriculture. Not surprisingly, the rural public – mostly farmers – account for more than three quarters of the total population. In fact, a significant number of smokers in China live in rural areas. Although smoking prevalence in urban populations is well documented, there is little information on smoking patterns among the rural public, and similarly, less is known about demographic characteristics of rural smokers in China. From both public health and tobacco control perspectives, it is vitally important to gather prevalence data of smoking in the rural population to help develop tobacco control policies and intervention and prevention strategies targeting this large population. To this end, using a cross-sectional design, the primary purpose of this study was to examine smoking patterns in male Chinese rural residents living in four geographic regions across China. A second purpose of the study was to assess sociodemographic correlates of smoking. On the basis of existing tobacco use literature in China [5.6], it was hypothesized that (a) there would be high smoking prevalence in the rural Chinese population, and (b) geographic regions and other salient sociodemographic characteristics, such as age, education, ethnicity, marital status, and income would be associated with smoking behavior.

## Methods

### The study's geographic area

The study's geographic area covered four regions in China: Southwest (Guiyang region), southeast (Subei region), northwest (Jinnan region), and north (Jinbei region). The decision to choose these geographic areas as the study sampling framework was based on the fact that these regions contained a range of low-to-high agriculture industry and population densities, features that allowed us to capture variations in smoking prevalence within and between regions.

### Study design and sampling

This is a cross-sectional study, which employed a multi-stage systematic sampling procedure to recruit study participants. In Stage 1, the four geographic regions mentioned previously were selected based on *a priori *decision. In Stage 2, within each geographic region, two counties were randomly selected. From these selections, 20% of rural villages within each county were sampled, and from these, 20% of local communities (the smallest unit in rural living areas) were chosen. In Stage 3, a list of family households was obtained through the local village government and administrative offices. From the households list, 20% of families were randomly selected and subsequently contacted. Residents who were eligible to participate in the study were male who were aged 15 years or older. In the case of two or more persons eligible in a family, the person with the birthday closer to the interview date was chosen.

### Procedures

Eligible individuals were interviewed at home by research assistants who were trained medical students from a local medical school. Upon receiving survey instructions explained by the assistants, participants were asked to fill out a survey questionnaire which lasted about 30 minutes. Each participant was given an opportunity to answer or clarify questions regarding survey or survey items and adequate time to complete the survey. Upon completion, participants were given ¥2.00 (equivalent of 25 cents in U.S. dollars) as a token of appreciation for participation. The Ethics Committee of the Zhejiang University School of Medicine approved the study and a written informed consent was obtained from each participant.

### Measures

Details of the measures used in this study are briefly described below. These measures have been used extensively in prior research on smoking involving Chinese populations with acceptable psychometric properties [5].

#### Smoking status

Participants were asked about their smoking status, including age of initiating smoking, current smoking frequency, average number of uses per day, types of tobacco smoked, and environmental tobacco exposure (ETS) of non-smokers. Smokers were defined as those who reported the use of any tobacco products, including cigarettes, cigars, and traditional Chinese hand-rolled and pipe cigarettes on a daily basic at the time of the study interview. Classification of occasional smokers were not considered in this study due to the difficulty of quantifying them accurately since smoking is almost always considered a daily activity among rural smokers [5.7]. The unit used to quantify the number of daily cigarettes, cigars, and hand-rolled cigarettes used is "cigarettes". For the sake of consistency in the unit of measurement, we also used "cigarettes" to quantify pipe use. In this case, one use of pipe cigarette was quantified as "one cigarette." Passive smokers in this study were defined as those who breathed in smoke exhaled by a smoker for more than 15 minutes per day.

#### Demographics

Participants' demographic information was collected as part of the overall survey. This included participants' residence, age, ethnicity, level of education, occupation, marital status, and annual personal income.

### Data analysis

Chi-square tests were used to calculate the smoking prevalence for the entire sample. Subsequent logistic regression analyses were carried out to test the hypotheses regarding smoking behaviors and their association with geographic and sociodemographic characteristics of the study participants. For the logistic analysis, the dependent variable was smoking status, operationalized as a binary response variable – smokers (those who reported daily smoking = 1) and non-smoking (those who reported no smoking = 0). Independent variables in the logistic regression equation were geographic regions, subjects' age, education, race, marital status, income, and job types. In the analyses, the referent groups for the sociodemographic variables were as follows: Subei for region, <25 years old for age, elementary or lower for education, never married for marital status, Han for ethnicity, farmer work for occupation, and <¥1000 (US$1 = ¥7.6) for income. Odds ratios (OR) were calculated and estimates are presented with 95% confidence intervals (CI). The odds ratios (ORs) are the probability of being a smoker. P values of 0.05 or less (2-tailed) were considered statistically significant. Data were analyzed using Statistical Analysis Software version 6.12 (SAS Institute Inc.). To adjust for clustering of the subjects within regions, weighted analyses are reported.

## Results

A total of 5,097 individuals were approached for participating in the study. Of those, 4,598 individuals were reached at the time of the contact and agreed to be interviewed, resulting in a response rate of 86.6% (4598/5097). Of the 4,598 participants, 4,414 individuals provided complete data that were reported in this study. Sociodemographic information of the study sample is presented in Table 1. The mean age for the study participants was 38.72 years (SD = 12.31). A vast majority of the participants were Han Chinese (97.9%), married (89.9%), had an education level that was lower than high school (85.2%), and an annual average personal income of ¥2,000 (US$1 = ¥7.6) or less (63.3%). Forty-two percent of the study participants reported having farm jobs. The demographic characteristics of the sample were similar to those of reported in the national population survey of Chinese male rural population [8].

**Table 1:** Sociodemographic characteristics of the study sample (N = 4,414)

Variable	N	% of total	Smokers (n, %)
Geographic region			
Subei	958	21.7	584 (61.0)
Jinnan	1375	31.2	915 (66.6)
Jinbei	1259	18.6	850 (67.5)
Guiyang	822	28.5	601 (73.1)
		*χ*^2^: 29.85, df=2, P<.001	
Age (years)			
< 25	759	17.2	459 (60.5)
25 – 34	981	22.2	675 (68.8)
35 – 44	1444	32.7	936(64.8)
45 -	1230	27.8	880(71.5)
		*χ*^2^: 30.53 df=3, P<0.01	
Education			
Elementary school	1864	42.2	1273 (68.3)
Middle school	1900	43.0	1281 (67.4)
High school	516	11.7	333 (64.5)
College or higher	134	3.0	63 (47.0)
		*χ*^2^: 27.06, df=3, P<0.01	
Martial status			
Never married	358	8.1	165 (46.1)
Married	3970	89.9	2731 (68.8)
Divorced/widowed	86	2.0	54 (62.8)
		*χ*^2^: 77.00, df=2, P<0.01	
Ethnicity			
Han	4320	97.9	2880 (66.8)
Minority	94	2.1	70 (74.5)
	*χ*^2^: 2.53, df=1, P>0.05		
Occupation			
Farm jobs	1871	42.4	1292 (69.1)
Floating jobs^#^	1533	34.7	1016 (66.3)
Non-farm related jobs	683	11.7	466 (67.4)
Others (no work capacity)	327	7.4	182 (55.7)
		*χ*^2^: 22.59, df=3, P<0.01	
Average annual income per person (¥)*			
< 1000	983	22.3	608(61.9)
1000–1999	1808	41.0	1233 (68.2)
2000–2999	891	20.2	597 (67.0)
3000–3999	377	8.5	289 (76.6)
>= 4000	355	8.0	223 (62.8)
		*χ*^2^: 31.53, df=5, P<0.01	

^#^Jobs that required going from place to place. * US$1 = RMB7.6

### Smoking behavior

Of the total 4,414 participants, 1,464 were classified as non-smokers. Of these, 1,252 (85%) were exposed to second hand smoking (i.e. passive smokers). A majority of the passive smokers reported being exposed to smoking in a labor field (46.1%), followed by locations such as home settings (23.7%) or public places (23.7%). Table 2 shows smoking behaviors of 2,950 (66.8%) smokers in the study sample. The mean age of smoking initiation was 21.70 years (SD = 9.44). The average use per day among smokers was 12.70 (SD = 7.99) and a majority of them reported smoking cigarettes (95%). Over 60 percent (n = 1908) of smokers reported daily smoking of more than 10 cigarettes. A majority of them (91%) reported buying cigarettes that were less than ¥10 per pack and the average monthly spending on buying cigarettes was ¥63.18 (SD = 49.94).

**Table 2:** Smoking behaviors among Chinese rural male residents (n = 2950)

	Total N	Percentage
Number smoked per day (cigarettes)		
< 10	1042	35.3
10 – 19	1022	34.6
20 or more	886	30.0
Starting age (years)		
< 18	238	8.1
18 – 20	1591	53.9
≥21	1121	38.0
Type of tobacco products usually smoked		
Cigarettes	2797	94.8
Cigar	10	0.3
Chinese Hand-rolled cigarettes	141	4.8
Chinese Pipes cigarettes	2	0.1
Cigarette price (¥/pack) (n = 2797)		
< 5	1034	37.0
5 – 9	1516	54.2
10 or more	247	8.8
Money spent on purchasing cigarettes per month (¥) (n = 2798)		
< 50	879	31.4
50 – 99	1519	54.3
100 or more	399	14.3

### Sociodemographic correlates of smoking

Results from the logistic regression analyses are shown in Table 3. Results indicated that participants living in the Jinbei were more likely to smoke than those living in Subei (OR = 1.94, 95%CI = 1.54–2.44), followed by the regions of Guiyang and Jinnan. The probabilities of smoking among male smokers aged 25–34 years and 45 and above were much higher than those under 25 years. The probability of smoking was significantly higher among ethnic minorities than Han Chinese (OR = 1.84, 95% CI = 1.29–2.62). Similarly, people who were married or divorced/widowed were 2.43 and 1.63 times, respectively, more likely to smoke than those who were unmarried. Participants with a college degree or higher were less likely to be smokers than those who had lower levels of education (OR = 0.51, 95%CI = 0.37–0.69). Those who were in the "other" work category were less likely to be smokers than those who engaged in farm work. Finally, the likelihood of being a smoker was significantly higher for those who reported higher annual personal income than those whose income was less than ¥1,000.

**Table 3:** Logistic regression results of sociodemographic factors associated with smoking (N = 4,414)

Category	Odds Ratio	95% C.I
Region		
Subei	1.00	--
Jinnan	1.37**	1.12–1.62
Jinbei	1.94**	1.54–2.44
Guiyang	1.39**	1.13–1.70
Age (years)		
< 25	1.00	--
25–34	1.29*	1.08–1.14
35–44	1.09	0.92–1.29
45-	1.30*	1.08–1.58
Education		
Elementary school	1.00	--
Middle school	0.94	0.83–1.07
High school	0.85	0.70–1.03
College or higher	0.51**	0.37–0.69
Martial status		
Never been married	1.00	--
Married	2.43**	1.99–2.98
Divorced/widowed	1.63**	1.11–2.38
Race		
Han	1.00	--
Minority	1.84**	1.29–2.62
Occupation		
Farmers	1.00	--
Floating jobs^#^	0.90	0.79–1.03
Non-farm related jobs	0.97	0.82–1.14
Others (no work capacity)	0.71*	0.57–0.89
Average annual income per person (¥)		
< 1000	1.00	--
1000–1999	1.33**	1.14–1.54
2000–2999	1.44**	1.21–1.72
3000–3999	2.07**	1.63–2.63
>= 4000	1.66**	1.23–2.08

^#^Jobs that required going from place to place.

*P < .05

**P < .001

## Discussion

In a sample of a Chinese rural male population, this study examined smoking prevalence and sociodemographic factors associated with smoking behavior. The prevalence of smoking is higher in this sample (66.8%) compared to that of urban residents (56.5%) but similar in smoking consumption (13.12, SD = 6.82) [9]. Also, a slightly higher proportion of rural population (91.9%) reportedly starting smoking at the age of 18 or older compared to the urban residents of the same age population (90.3%) [9]. Overall, the finding generally reflects higher prevalence of smoking in rural communities compared to urban communities in China [10,11]. While the underlying mechanism(s) for the rate discrepancies between rural and urban smokers remains to be investigated, some have indicated cultural and social dimensions of lifestyle that may account for the rural-urban differences [5].

Smoking behavior was found to be associated with a number of sociodemographic characteristics in this study. First, there was a significant variation across the four study geographic regions, a finding that is consistent with those reported with urban populations [9]. The finding may reflect the fact that there are significant regional differences in lifestyle and cultural standards across mainland China [5]. For example, the finding that residents living in the Subei region were less likely to smoke than those living in other geographic regions may be due to the fact that the Subei region is the most developed geographic area of all four regions. Therefore, it is conceivable that people's lifestyle and beliefs may be influenced by the social and economic developments in the area. Thus, being residents living in socially and economically well-developed areas or regions may provide them with an environment in which a healthier lifestyle is promoted. It is interesting to note the higher probability of smoking (OR = 1.84, 95%CI = 1.29–2.62) and smoking consumption (mean = 18.00, SD = 7.48 for the minority, mean = 12.52, SD = 8.08 for Han Chinese, respectively) among minority Chinese compared to the Han Chinese. A complementary analysis indicated that most minority Chinese male rural residents in the study were living in Guiyang, a remote and underdeveloped region in China.

Consistent with prior research [6,10,11], age was another demographic factor that was related to smoking, with the oldest age category (i.e., 45 years or older) being more likely to engage in smoking. Also consistent with the literature [9,10] is the finding that married or divorced/widowed men were more likely to smoke than unmarried persons, a finding that is in contrast to those that reported no difference by marital status [11,12]. Similarly, participants with a college degree or higher were less likely to smoke compared to those who had lower levels of education. While a majority of participants in the study reported engaging in farm work (42.4%), this is also the subgroup of individuals who were most likely to smoke when compared to those who reported engaging in unclassified work (i.e., "other"). Finally, participants with higher annual income were more likely to smoke than those with lower income. There is a general trend showing that smoking consumption increased with the increase of annual average income, a finding which is in contrast to some studies that found no difference among different income status groups [11,13]. However, it appears that a majority of smokers in this study (91.2%) purchased packages of cigarettes that were under ¥10 (US$1.3); the proportion who purchased cigarettes packs of under ¥10 was lower in urban smokers (52.5%) [9]. The average monthly cigarette related expenditure was ¥62.1 (US$8.2). Current estimates in China indicate a monthly farmer family income of ¥788.8 (US$103.7) in 2003 [14], reflecting that about 7.9% of family income was spent per person on cigarette smoking among tobacco users.

There are at least two notable limitations associated with the current study. First, the cross-sectional study design precludes any causal inference to be drawn from these findings. Currently, there are no longitudinal studies conducted in China to track smoking prevalence over time. Future research is needed to collect both cross-sectional and longitudinal smoking surveillance data in both rural and urban communities. In addition, community-health promotion and policy-level data is needed to allow better understanding of how smoking is related to or influenced by community-level health regulations or anti-smoking campaigns. Second, only male individuals were observed in this study, which may limit its generalizability to the overall rural population. However, we purposefully selected the male population because male residents constitute a major smoking population in the rural communities of China.

With these limitations in mind, the current study also has a number of strengths. First of all, this was the first study that focused exclusively on the Chinese farmer population, which makes it unique among all other studies that have been conducted in China to date. In addition, unlike other Chinese studies, this study used the WHO definition of smoking which means that the current results may be compared directly to other smoking prevalence studies, both locally and internationally. Finally, the large sample size, which resulted from the use of four large but distinct geographic regions as a sample framework, is also a strength allowing a broad representation of the sample population of rural China. It should be noted that we used geographic regions rather than provinces as a sampling framework. This maximizes the between-region differences with respect to geographic, cultural, and social-economic characteristics, and therefore allows us to capture a sample that is homogeneous within regions and heterogeneous among regions.

## Conclusion

The current study contributes to the extant smoking literature by documenting high prevalence of smoking among male residents living in rural Chinese communities. Findings also indicate important differences in tobacco use among users sampled across four geographic regions, as well as several demographic characteristics that are associated with smoking behaviors. Findings from this study collectively suggest that both government and local health agencies need to consider tobacco control policies and develop intervention and prevention strategies to lower the overall prevalence of the smoking epidemic in rural China.

## Competing interests

The authors declare that they have no competing interests.

## Authors' contributions

TY originated the study, supervised all aspects of its implementation, performed data analysis, and wrote the article. ZW, XF, YW and XW assisted in data collection. FL contributed to the interpretation and writing of the article. XY assisted with data analyses and provided input on drafts of the manuscript. ASA assisted with the study design, analytic plan and revising the final draft of the manuscript. All authors reviewed drafts of the manuscript and approved the version to be published.

## Pre-publication history

The pre-publication history for this paper can be accessed here:



## References

[B1] World Health Organization (1999). Combing the tobacco epidemic: In world health report.

[B2] Liu BQ, Peto R, Chen ZM, Boreham J, Wu YP (1998). Emerging tobacco hazards in China: Retrospective proportional mortality study of one million deaths. BMJ.

[B3] Niu SR, Yang GH, Chen ZM, Wang JL, Wang GH (1998). Emerging tobacco hazards in China: Early mortality results from a prospective study. BMJ.

[B4] Peto R, Chen ZM, Boreham J (1999). Tobacco the growing epidemic. Nature Med.

[B5] Yang T, Yang T (2007). Smoking. Health Behavior theory and research.

[B6] Hong G, Wang D, Wang H, Wang F, Zhu L, Cai J (2007). Prevalence survey of smoking behavior and socioeconomic status among rural residents in Anhui province. Chin Rural Health Service.

[B7] World Health Organization (1998). Guidelines for controlling and monitoring the tobacco epidemic.

[B8] National bureau of statistics of China (2004). China population statistics yearbook.

[B9] Yang T, Huang H (2003). An epidemiological study on stress among urban residents in social transition period. Zhonghua Liu Xing Bing Xue Za Zhi.

[B10] Liu ZM, Li YJ, Li X, Li G, Shi TX, Liu XJ (2006). An Survey of Smoking Behaviors in Liaoning province. Chinese Chronic Disease Prevention and Control.

[B11] Yang G, Ma J, Liu N, Zhou J (2002). Smoking and passive smoking in Chinese. Chin J Epidemiol.

[B12] Unger JB, Shakib S, Cruz TB, Hoffman BR, Pitney BH, Rohrbach LA (2003). Smoking behavior among urban and rural Native American adolescents in California. Am J Prev Med.

[B13] Chen X, Li X, Stanton B, Fang X, Lin D, Cole M, Liu H, Yang H (2004). Cigarette smoking among rural-to-urban migrants in Beijing, China. Prev Med.

[B14] Zeng P (2003). Report on China's national economic and social development for 2003.

